# The Impact of Internalizing Symptoms on Impairment for Children With ADHD: A Strength-Based Perspective

**DOI:** 10.1177/10870547221115874

**Published:** 2022-08-03

**Authors:** Sarah C. Bethune, Maria A. Rogers, David Smith, Jess Whitley, Michael Hone, Natasha McBrearty

**Affiliations:** 1 University of Ottawa, ON, Canada; 2 Carleton University, Ottawa, ON, Canada; 3 Crossroads Children’s Mental Health Centre, Ottawa, ON, Canada

**Keywords:** functional impairment, internalizing, strengths, ADHD

## Abstract

**Background/Purpose:**

This study aims to investigate the influence of internalizing symptoms on functional impairment for children with ADHD, and whether child strengths and parenting strengths have moderating effects on this relationship.

**Methods:**

Participants included 209 children with ADHD and their caregivers seeking mental health services between the ages of 5 and 11 years. To examine the moderating effects of parenting and child strengths, ordinary least squares regression models were tested using the PROCESS macro for SPSS (v3.5).

**Results:**

Results suggest that levels of internalizing symptoms influence functional impairment in children with ADHD. Child strengths moderate the relationship between internalizing symptoms and functional impairment when internalizing symptoms are medium to high.

**Conclusion:**

Findings from this study demonstrate that facilitating child strengths can help moderate functional impairment for children who experience ADHD and internalizing symptoms.

ADHD is one of the most prevalent and well researched childhood psychological disorders (Sciberras et al., 2017). In recent years, research in this area has focused heavily on functional impairment, as children with ADHD typically experience challenges beyond the core symptoms described in the Diagnostic and Statistical Manual for Mental Disorders, 5th ed. (DSM-5; APA, 2013). While there is some evidence suggesting that a relationship exists between symptom severity and functional impairment, many studies have demonstrated that these factors are not completely overlapping (Barkley, 2006; Healey et al., 2011). Although functional impairment is required for diagnosis of ADHD, the symptoms themselves cannot be used as a measure of functional impairment. (Willcut et al., 2012).

Many children with ADHD experience issues with academic, social, and family functioning (Meltzer et al., 2003; Pelham et al., 2005). Some examples include difficulty completing schoolwork, low academic achievement, poor social and communication skills, lack of friendships, and issues with family cohesion (A. Becker et al., 2006; Dupaul, 2007; Escobar et al., 2005; Klimkeit et al., 2006; Wu & Gau, 2013). Functional impairments are one of the primary reasons for clinical referral, meaning that they are a source of distress for both children and their families (Barkley, 2006; K. D. Becker et al., 2011).

To effectively treat children with ADHD in a clinical setting, it is essential to gain a better understanding of why children experience various levels of functional impairment, despite sharing the same diagnosis of ADHD. This study will examine the role of comorbid internalizing symptoms and various risk and protective factors that may be influencing functional impairment across several domains.

This research is conceptualized using the cumulative risk model, which suggests that psychological health is influenced by the number of risk factors that are present in an individual’s life (Rutter, 1981); and transdiagnostic models, proposing that psychological distress should be perceived dimensionally rather than categorically (Achenbach, 1966; Krueger & Eaton, 2015).

## ADHD and Internalizing Symptoms

Internalizing symptoms encompass a variety of difficult emotions, thoughts, and beliefs that are directed inward by the individual (Merikangas et al., 2010; Pfiffner & McBurnett, 2006). They can be experienced through emotional distress, frustration, feelings anxiousness or sadness, and somatization among others (Merikangas et al., 2010). Internalizing problems in children have often been associated with sleep difficulties, peer problems, social isolation, low self-esteem, low academic achievement, higher rates of suicidality, and family conflict (S. P. Becker et al., 2012; Breaux et al., 2020; Karustis et al., 2000; Loe & Feldman, 2007; Pfiffner & McBrunett, 2006). Previous literature suggests that children with ADHD experience a high rate of comorbid internalizing issues (Connor & Ford, 2012; Melegari et al., 2018). Moreover, it has been found that individuals with ADHD and internalizing symptoms often experience higher levels of functional impairment compared to their counterparts with exclusively symptoms of ADHD (Bishop et al., 2019). These findings highlight the importance of investigating the impacts of comorbidity on child development. Moreover, there is some evidence suggesting that internalizing symptoms can change the expression of ADHD. For example, comorbid ADHD and anxiety has been associated with further inattentiveness, issues with working memory, and less impulsivity (Melegari et al., 2018). Understanding the comorbidity between several disorders can help clinicians better comprehend the impact and developmental trajectories of the disorders as well as formulate appropriate treatment plans (Caye et al., 2016; Connor & Ford, 2012).

## Risk and Protective Factors

There are several factors that are likely to influence the relationship between internalizing symptoms and level of functional impairment in children with ADHD. Risk factors increase the chances of undesirable outcomes (Appleyard et al., 2005), whereas protective factors buffer against risk and are expected to result in resiliency (Climie & Mastoras, 2015). Examining risk and protective factors provides greater insight into the trajectories of ADHD. Furthermore, this perspective emphasizes factors that contribute to adjustment and well-being, instead of solely problematic symptoms (Climie et al., 2013). Having a more balanced viewpoint provides opportunity to create strength-based interventions that may facilitate well-being and decrease functional impairment (Climie & Mastoras, 2015). This study will be focusing on family and child factors in relation to functional outcomes. Parenting practices have been found to influence the manifestation of functional impairment (Pressman et al., 2006), while certain child characteristics have been found to moderate risk for children with ADHD (Dvorsky & Langberg, 2016; McCrimmon et al., 2018).

### Family Factors

#### Negative parenting practices

In the literature, negative parenting practices often refers to authoritarian parenting, inconsistent discipline, negative parental control, harsh punishments, and unresponsive parenting (Ellis & Nigg, 2009; McRae et al., 2020). These parenting practices have commonly been associated with poor outcomes for children, especially those with ADHD (Kaiser et al., 2011). For example, parents who use inconsistent discipline are more likely to have children who meet the criteria of an ADHD diagnosis, regardless of comorbid behavioral issues such as conduct or oppositional defiant disorder (Ellis & Nigg, 2009). Additionally, negative parenting practices appear to play a role in the development of comorbid disorders. Children who are exposed to negative parenting practices such as harsh, disengaged, or inconsistent parenting, are more likely to develop further externalizing symptoms and internalizing symptoms (Aunola & Nurmi, 2005; Deault, 2010; Kaiser et al., 2011; McRae et al., 2020; Pfiffner & McBurnett, 2006).

Not only are negative parenting practices linked to additional symptoms, they are also associated with lower levels of child functioning. A study by Kaiser et al. (2011) demonstrated that parenting practices have a significant impact on the child’s social skills regardless of the severity of their ADHD symptoms. Likewise, children who experience negative parenting are more likely to demonstrate aggressive behaviors toward peers (Kaiser et al., 2011). As such, it is expected that they will experience additional peer rejection and social difficulties (Fenesy et al., 2019).

#### Positive parenting

Positive parenting is described as authoritative, warm, supportive, consistent, and responsive to the child’s needs (McRae et al., 2020). Positive parenting practices have been found to increase resilience in children with ADHD (Arsenio & Ramos-Marcuse, 2014; Dvorsky & Langberg, 2016; Schei et al., 2015). For example, parents who engage in positive parenting are more likely to have children with less severe symptoms of ADHD and fewer internalizing and externalizing behaviors (Healey et al., 2011; McRae et al., 2020). Positive parenting is associated with overall wellbeing, less aggression and defiance, better problem solving, and emotion regulation skills (Arsenio & Ramos-Marcuse, 2014; Demaray et al., 2005; Mastoras et al., 2018). Additionally, research demonstrates that positive parenting predicts stronger social functioning in children with ADHD (Kaiser et al., 2011). It is suggested that these practices are particularly relevant to developmental outcomes during early childhood (Dvorsky & Langberg, 2016). By understanding the role of positive parenting practices, clinicians will be better able to create strength-based treatment plans that may highlight or facilitate these skills.

### Child Factors

While it is clear that parenting practices influence child outcomes, there is less research addressing individual characteristics of the child in relation to their level of functioning.

#### Child protective factors

Much of the literature on individual characteristics and resiliency for children with ADHD focuses on social functioning (Dvorsky & Langberg, 2016; McCrimmon et al., 2018; Ray et al., 2017). In fact, perceived peer acceptance, the ability to foster positive peer relationships, participation in social activities, and the ability to share emotional intimacy are protective factors that buffer against functional impairment (Dvorsky & Langberg, 2016; McCrimmon et al., 2018; Ray et al., 2017). A study conducted by Dvorsky and Langberg (2016), demonstrated that children with ADHD who felt accepted by their peers were more likely to have higher grades in school and reduced symptoms of inattention and depression. Feelings of self-efficacy, autonomy, competence, and positive self-perception were also found to be protective factors for children with ADHD (Dvorsky & Langberg, 2016; Hechman, 1991; McCrimmon et al., 2018). Moreover, a positive view of oneself has been linked to the ability to adapt to life stressors and in turn, protect against the development of internalizing symptoms such as depression (Dvorsky & Langberg, 2016). Finally, a study conducted by McCrimmon et al. (2018), suggested that interventions for children with ADHD should include facilitating emotional intelligence (EI), as EI may contribute to building resilience. Examples of EI include self-awareness, decision making, and communication skills.

## Objectives of the Current Study

Given that ADHD is one of the most prevalent psychological disorders among children, it is essential to explore the complexities of the disorder and how it affects their day-to-day lives (Sciberras et al., 2017). Research on functional impairment has become increasingly relevant, as children with ADHD often experience additional challenges that are not directly associated to their diagnosis (Healey et al., 2011). In line with the transdiagnostic model, children with ADHD report experiencing further internalizing and externalizing issues. The vast body of research in this area demonstrates that comorbid conditions are likely to pose a barrier to positive outcomes (S. P. Becker et al., 2015; Breaux et al., 2020; Ter-Stepanian et al., 2019).

Decades of research has focused on the detrimental impact of family stress and negative parenting for children with ADHD (Kaiser et al., 2011; McCrae et al., 2020). Contrarily, there is evidence suggesting that positive parenting practices can facilitate strengths and serve as a protective factor against impairment (Healey et al., 2011; McCrae et al., 2020). There is some research demonstrating that individual strengths aid the development of resilience in children with ADHD, however studies in this area have been limited (McCrimmon et al., 2018).

While numerous studies have considered these variables, no study has investigated them together in this context. Guided by the cumulative risk model and transdiagnostic models, this study aims to gain a more comprehensive understanding of functional impairment in children with ADHD in a clinical sample, particularly, to understand the effects of comorbid internalizing symptoms on levels of impairment. This study adopts a strength-based perspective by examining individual and family characteristics that have the potential to serve as protective factors, moderating the risk of functional impairment. Examining protective factors provides a perspective that is less frequently discussed in the literature and will help inform strength-based interventions. The proposed research will explore the following questions: Is there a relationship between internalizing symptoms and functional impairment for children with ADHD? Do family strengths and child strengths moderate this relationship?

## Method

### Participants

Data derive from a pre-existing database of measures administered by a not for profit, urban, community mental health center in a city in eastern Canada, which provides services at no cost to clients. Clients who utilize these services are self-referred or referred from various community agencies such as Children’s Aid Society, school-based mental health services, hospitals, the Arson Prevention Program for Children or the police. Children who utilize these services are often identified by their parents and or their teachers as experiencing emotional, behavioral, or social difficulties.

The sample for this study included 250 participants whose parent or guardian reported a diagnosis of ADHD determined by a professional in the community. In order to be included in the analysis, participants’ parents had to report a diagnosis of ADHD and meet the cut off score of 7 out of 10 on the hyperactivity scale of the SDQ. Those who did not have been excluded from the analysis. The original sample was 250 participants. After screening for inclusion criteria, the current sample is 209 children receiving mental health services.

### Data Collection and Measures

This study applies secondary data collected by a community mental health center in eastern Canada, gathered from clinicians and parents between April 2017 and January 2020. All participants partake in an intake interview and are administered a battery of scales upon registration with the center. This consists of basic demographic data, information regarding the presenting problem, the Child and Adolescent and Childs Needs and Strength Questionnaire (CANS), and the SDQ. The SDQ is completed by the parent and the CANS is completed by the clinician. Diagnostic information is collected by the center through two pathways. During the initial intake interview at the center, clinicians asked parents whether their child had been diagnosed by a professional in the community. This diagnosis was later supported using the cut off score of 7 out of 10 on the hyperactivity scale of the SDQ (Algorta et al., 2016; Cuffe et al., 2009). Children who did not meet both criteria were excluded from the study by researchers.

#### Strengths and Difficulties Questionnaire

The SDQ is an internationally used, brief screening measure assessing psychosocial difficulties and strengths in children and adolescents aged 4 to 16 years old (Goodman, 1997; Stone et al., 2010). This study utilizes the parent reported version of the SDQ which has been found to have acceptable internal consistency and predictive value regarding the child’s wellbeing over time (α = .66; Biel et al., 2015; S. P. Becker et al., 2015; van Widenfelt et al., 2003). The SDQ consists of 25-items divided into five subscales and is scored on a 3-point Likert scale ranging from 0 “not true” to 2 “certainly true.” The impact supplement score is used to measure the overall impact of psychosocial difficulties on the child’s life. The parent rated version of the SDQ includes five items that are scored on a Likert scale ranging from 0 “Not at all,” 0 “Only a little,” 1 “A medium amount,” and 2 “A great deal” with a maximum overall score of 10. Items focus on the child’s overall distress, interference with home life, friendships, classroom learning, and home activities (Stone et al., 2010). The impact score on the SDQ was intended to measure functional impairment and several studies have used this score to understand level of functioning in children (Biel et al., 2015; Goodman, 2001).

The SDQ has satisfactory reliability and validity across samples from various countries including Canada (Aitken et al., 2015; Goodman 2001; Marquis & Flynn, 2009; Yao et al., 2009). There is evidence of internal consistency within the subscales (α = .80–.95) and the total impact score (α = .80; Aitken et al., 2015; Goodman, 2001). The interrater agreement is above average. The test-retest reliability for the total difficulties scale with an 8-week interval is satisfactory(*r* = .71), as is the test-retest reliability for the emotional problems scale (*r* = .70; Goodman, 2001; Yao et al., 2009). The SDQ has demonstrated concurrent validity with the Rutter Questionnaire and the Achenbach Child Behavior Checklist (CBCL; Goodman & Scott, 1999; Stone et al., 2010). Construct validity remains the same regardless of gender, age, or ethnicity (Stone et al., 2015).

The current study utilizes the emotional problems subscale to examine internalizing symptoms. The impact supplement score is used to examine the child’s level of functional impairment across multiple areas and is the outcome variable for this study. Furthermore, the hyperactivity scale is used to strengthen the reliability of the child’s existing ADHD diagnosis. The hyperactivity scale consists of two items relating to hyperactivity, two items assessing inattention, and one item pertaining to impulsivity. Previous studies have found that the hyperactivity sub score on the parent version of the SDQ is a statistically valid tool for differentiating individuals with ADHD from those without, irrespective of age or gender (Algorta et al., 2016; Hall et al., 2019; Rimvall et al., 2014). This remains true regardless of whether the individual has comorbid disorders such as ODD or other externalizing symptoms (Algorta et al., 2016). As such, the SDQ is able to discriminate between ADHD and other psychological disorders and can be justifiably used to confirm existing diagnoses of ADHD in this sample.

#### The Child and Adolescent Needs and Strengths Questionnaire

The CANS assessment is designed to evaluate the needs and strengths of children and families across multiple areas. It is a communimetric tool, meaning that it can be adapted to fit the needs of the specific agency, while remaining valid and reliable (Lyons, 2009). In this study, a short form version of the CANS was used. This version contains 28 items that address the needs of the center directly (Anderson et al., 2003). Moreover, it highly resembles the CANS-MH, a measure designed to be administered in community mental health agencies (Anderson et al., 2003). The CANS is intended to be used in a clinical setting to determine level of action required on behalf of the clinician and guide treatment options (Anderson et al., 2003). However, the CANS-MH is also a reliable measure of psychosocial needs and strengths when used by researchers (Lyons et al., 2000). In fact, archival reviews have been found to be valid and reliable when investigating characteristics of children with mental health difficulties (Burchard & Schaefer, 1992). The CANS has demonstrated evidence of reliability (0.90), face validity and construct validity (Lyons et al., 2004).

The CANS is completed by clinicians based on information provided by caregivers during the interview. The questionnaire is scored on a 4-point Likert scale. For parenting strengths, “0” represents no evidence, “1” represents watch/prevent, “2” represents act, and “3” represents a need for immediate or intensive action. For child strengths, “0” represents centerpiece, “1” represents useful, “2” represents identified, and “3” represents not yet identified. In both cases, the lower the number, the more strengths the child or parents possess.

Prior to this research, a factor analysis was conducted on the 28 items of the CANS as a part of a different project. The purpose of the factor analysis was to create composite variables that accurately reflect parenting and child strengths. Results suggested that the 28 items loaded into four main domains, 2 of which are being used for this study; parenting (problem solving, parental responsiveness, discipline skills, impact of one’s own behavior on child, parent/child relationship, knowledge of child needs, family stress, and ability to communicate) and child strengths (adaptability to change, self-expression, and positive peer relationships and family).

These composite variables were adjusted to reflect how strongly they fit together conceptually and statistically. In this study, the “family” item was dropped from child strengths variable, as it did not fit what we were trying to measure conceptually. Particularly, we were interested in the intra-individual aspects of child strengths. The “family stress” and “ability to communicate” items were removed from the parenting strengths variable, as doing so increased the reliability of the variable.

Child strengths, including adaptability to change, self-expression and positive peer relationships and parenting strengths, encompassing problem solving, parental responsiveness, discipline skills, impact of one’s own behavior on child, parent/child relationship, and knowledge of child needs will be utilized in this study. Cronbach’s alpha for the three child strengths items and the six parenting strengths items were .65 and .88 respectively.

### Data Analysis

Descriptive statistical analyses were conducted using SPSS to better understand the characteristics of this sample. In order to examine the moderating effects of child strengths and parenting strengths, ordinary least squares regression models were tested using the PROCESS macro for SPSS (v3.5; Hayes, 2018; Hayes & Rockwood, 2017). This tool was selected, as it automatically centers the independent variables, creates interactions terms, and produces simple slopes for continuous moderator variables (Saidi & Branscum, 2019). In this case, simple slopes were examined at the mean and ±1*SD* for significant moderators. Internalizing symptoms will be used as an independent variable, parenting and child strengths are the moderating variables and overall impairment is the dependent variable. Significance is determined at the .05 probability level (Lin et al., 2020). The analysis includes an examination of two models: Internalizing symptoms, child strengths, and functional impairment and internalizing symptoms, parenting strengths, and functional impairment.

## Results

### Demographic Data

Participants were between the ages of 5 and 11 years (*M* = 8.15 years, *SD* = 1.68) with an ADHD diagnosis. Most of the sample was male (75%), Caucasian (85.6%), and spoke English as their first language (91.4%). This community mental health center tends to serve low-income families. About 21% of the sample had a total family income of less than $60,000 and were considered low income (*N* = 44). It should be noted that approximately half of the sample did not disclose their income. However, the 2019 to 2020 annual report provided by the community mental health center suggests that 28% of families who utilize their services have a combined family income of less than $30,000, 16% had a total family income of between $30,000 and $59,000. Although these statistics do not reflect the sample directly, they are indicative of clients at this facility (Crossroads Children's Mental Health Centre, 2020). The most recent census conducted in the city that the data was collected, suggests that the median family income is meaningfully higher than the total family income seen at this community mental health center (Ottawa, 2016). Most participants were diagnosed with the combined presentation of ADHD (90.9%, *N* = 190) and parents reported that 47% of the sample had at least one comorbid disorder (*N* = 98). Of these 98 participants, 35% reported disruptive or conduct disorder, 30% reported an anxiety disorder, 13% reported autism spectrum disorder, 12% reported depressive disorder, 12% reported a specific learning disorder, 7% reported trauma related disorder, 3% reported obsessive compulsive disorder, 3% reported an intellectual disability, 3% reported tic disorder, and 1% reported communication disorder. Approximately 50% of the sample was taking medication at the time that data was being collected.

### Preliminary Analyses

Table A1, included in Appendix A, demonstrates the means, standard deviations, ranges, and correlations between variables. The level of functional impairment experienced by participants was positively significantly correlated with their internalizing symptoms (*r* = .25) and need for child strengths (*r* *=* .23). Results indicate a weak positive correlation between child and parenting strengths (*r* = .17). It should be noted that low scales indicate no clinical concern, whereas, rising scores indicate potential clinical issues.

### Model 1: Internalizing Symptoms and Parenting Strengths

We first investigated whether there was a relationship between internalizing symptoms and level of functional impairment experienced by children with ADHD and if parenting strengths moderate this relationship. Results suggests that in this model, internalizing symptoms significantly influenced functional impairment (β = .22, *t* = 3.67, *p* < .01). Parenting strengths was nearing significance as a predictor for functional impairment. (β = .10, *t* = 1.89, *p* = .059). There was no significant moderation effect (β = −.02, *t* *=* −1.01, *p* = .30). Despite an insignificant moderation effect, this model was significant (*R*^2^ =0.08, *F*(3,205) = 5.98, *p* < .01).

### Model 2: Internalizing Symptoms and Child Strengths

We next investigated whether there was a relationship between internalizing symptoms and level of functional impairment experienced by children with ADHD and whether child strengths moderate this relationship. Results suggested that internalizing symptoms significantly influenced functional impairment (β = .19, *t* = 3.26, *p* = .001). Child strengths was a significant predictor of functional impairment (β = .27, *t* = 2.96, *p* = .003). There was a significant moderation effect (β = .08, *t* = 2.19, *p* = .03). This model was significant (*R*^2^ = .12, *F*(3,205) = 9.39, *p* < .01). Table B1 included in Appendix B, provides a visual representation of results.

**Figure B1.**
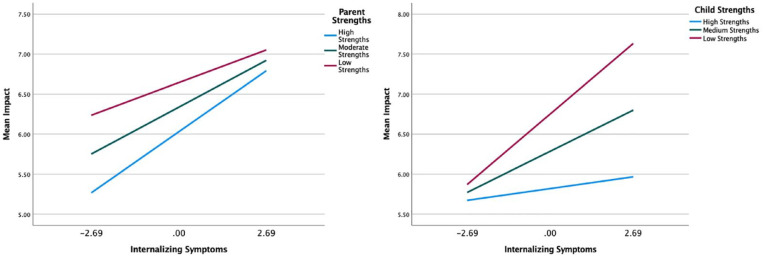
Moderation analysis for parent strengths and child strengths with internalizing symptoms as a moderator.

To better interpret the nature of the moderated relationship between internalizing symptoms and functional impairment, simple slopes were examined at the mean at ±1*SD* for child strengths. This means that participants were separated in to three categories: Those who scored below the mean (low internalizing symptoms), around the mean (moderate internalizing symptoms), and above the mean (high internalizing symptoms). Results suggested that child strengths have an effect when internalizing symptoms are moderate (β = .19, *t* = 3.26, *p* < .01) and high (β = .32 *t* = 3.89, *p* < .01). Figure B1, included in Appendix B, depicts the plotted simple slops for both models 1 and 2.

## Discussion

The purpose of this study was to investigate the relationship between internalizing symptoms and functional impairment in children with ADHD. Additionally, we wanted to explore whether child strengths and parenting strengths influence the impacts of internalizing symptoms, thus reducing levels of functional impairment. Results suggest that that the higher the level of internalizing symptoms, the more functional impairment the child is likely to experience. Results from this study are in accordance with current literature suggesting that children with ADHD who have internalizing comorbidities experience higher levels of functional impairment (Bishop et al., 2019).

In this sample, parenting strengths did not moderate the association between internalizing symptoms and functional impairment. Therefore, parenting practices, whether positive or negative did not have a significant effect on the association between internalizing symptoms and children’s functioning. The relationship between parenting strengths and functional impairment was nearing significance, meaning that parenting practices have a marginal impact on functioning, however the impact does not differ based on the level of internalizing symptoms. Based on the existing literature, it is surprising that parenting strengths did not play a more prominent role in contributing to both the child’s level of functioning and influencing the association between the independent and dependent variables. For example, there is a clear consensus in the literature that parenting practices can be predictive of outcomes for children with ADHD (Kaiser et al., 2011; Wustner et al., 2019; Dvorsky & Langberg, 2016; McRae et al., 2020). There are several reasons that this mild discrepancy between existing literature and our current results could have occurred. As previously mentioned, data in this study are collected in an intake session upon meeting the family for the first time. It is possible that clinicians have not had enough time with the family to get an accurate impression of the parents’ skills. In fact, the mean for parenting strengths is quite low. As mentioned previously, low scores indicate less of a clinical concern. Given the sample, it would be expected that the baseline clinical concern or need for parenting and child strengths would be high. Parents in this sample may have high levels of parenting strengths. Alternatively, there could be an issue with clinicians’ evaluation of parenting strengths. Previous literature examining parenting skills use primarily self-report measures and observational methods (Deault, 2010; Kaiser et al., 2011; Park & Walton-Moss, 2012). Therefore, it is possible that the difference in measurement yields varying results.

Child strengths was a significant moderator in the relationship between internalizing symptoms and functional impairment. Follow up analyses indicate that child strengths exert an increasing influence on functional impairment as internalizing symptoms become moderate and high. Specifically, children who have a high level of strengths, experience less overall impairment, despite having high internalizing symptoms. Alternatively, children who demonstrate low level of strengths, are more likely to experience functional impairment across several domains. These results indicate that child-level strengths can protect against functional impairment and should be considered when treating the individual. It should be noted that strengths are conceptualized differently depending on the study. For example, some studies have considered variables such as peer relationships, emotional intelligence, and self-perceptions as child strengths (Dvorsky & Langberg., 2016; McCrimmon et al., 2018; Ray et al., 2017), whereas this study focuses on positive peer relationships, self-expression, and adaptability to change. Past literature in combination with the results of this study demonstrate that regardless of how child-level strengths are conceptualized, there is growing evidence that they have a protective value for children with ADHD. As this area of research is relatively small, future research should consider continuing to investigate ADHD from a strength-based perspective.

It is important to note that results from this study cannot be generalized, as data is collected from a clinical sample. Families who seek services from this community mental health center are more likely to be experiencing high levels of impairment and additional comorbidities. Although these results are not representative of children with ADHD in the general population, they can help understand the experiences of children and their families who seek professional services. Although these results are directly relevant to this population, it could be speculated that results would be similar for children who do not have ADHD, as parenting and child strengths may be helpful for all children, not solely those with a specific diagnosis.

### Contributions to Clinical Practice

Functional impairment is one of the primary reasons that families seek mental health support (K. D. Becker et al., 2011). Much of the research in this area has focused on the challenges faced by children with ADHD and their families. This study provides a more balanced perspective by examining both the challenges that children face, along with the potential strengths that can play a role in their outcomes. Understanding the child’s experience from this perspective can help clinicians, teachers, and parents shift their attention to seeking opportunities for growth within the child and nurturing their strengths. Additionally, results from this study highlight the importance of conceptualizing a presenting issue from a strength-based lens and utilizing strength-based interventions. Previous research suggests that children with ADHD in a classroom setting who learn how to identify and utilize their strengths, are more likely to experience enhanced engagement, sense of self-worth, and motivation (Goldstein et al., 2013). Helping teachers, parents, and clinicians understand both the deficits associated with ADHD and the strengths of the child can promote resilience and an overall sense of wellbeing (Climie & Mastoras, 2015).

### Limitations and Strengths

This research was done in the context of a community agency. There are some inherent limitations attached with conducting research in this setting. This study utilized secondary data that had already been collected as a part of the intake process at a community mental health center. Clinicians were collecting data for clinical, not research purposes. Accordingly, researchers did not have autonomy over measures used or the data collection process. It would have been beneficial to have a rigorous interview with the family and more thorough measures. For example, rather than relying on a parent reported diagnosis of ADHD and ratings on the SDQ, researchers could have also conducted their own assessment of ADHD, which is common in most ADHD research (Fenesy et al., 2019). Variables in this study are quite broad. Researchers were unable to separate scores on the four items of functional impairment (social, family, leisure, and classroom). It may have been interesting to see if children experience more functional impairment in certain domains rather than others. Additionally, there are some disadvantages of using the same informants (i.e., parents) to measure both the independent and dependent variables in this study. Any bias present in the rating of internalizing symptoms, will also be present when rating functional impairment. This could account for an inflated correlation between internalizing symptoms and functional impairment.

Despite the limitations of working with data collected for clinical purposes by a community agency, there are also several advantages. Firstly, this study has ecological validity. Utilizing data from a community agency allowed us to understand individuals in a clinical setting who may have lower SES, more comorbidities, and complex challenges. This is important, as it is common for children with ADHD to experience several comorbid symptoms. Additionally, it allows us to better understand the heterogeneous nature of ADHD and cumulative risk (Pelham et al., 2005; Rutter, 1981). Using secondary data allows for a large sample size, increasing the reliability of results. There are some advantages to having multiple informants in this study. Having a different informant for the moderator variables (i.e., the clinician) provides a different perspective, potentially reducing bias, and creating a more accurate depiction of the child’s experience.

### Future Questions

Future research should focus on individual child strengths more closely to examine if any particular strengths are more impactful than others. Additionally, it could be interesting to examine child strengths in different contexts, to better understand the extent of the generalizability of this result. For example, it is possible that child strengths play a role in moderating negative outcomes in other relationships. It is important to further investigate which interventions amplify child strengths and whether this influences functional impairment. Since there was a high percentage of participants with comorbidities in this sample, future studies should consider the presence of other disorders in outcomes for children with ADHD. Lastly, it may be interesting to investigate whether results would be similar if this study was conducted using a non-ADHD sample, or whether there is something in particular about the experience of having ADHD that led to these findings.

## Appendix A

**Table A1.** Descriptive Statistics and Correlations.

Variables	*n*	*M*	*SD*	Minimum	Maximum	1	2	3	4
1.Internalizing symptoms	209	5.24	2.69	0	10	—	.25**	.15*	−.02
2.Functional impairment	209	6.34	2.37	0	10		—	.23**	.11
3.Child strengths	209	5.54	1.71	0	9			—	.17*
4.Parenting strengths	209	5.83	2.91	0	18				—

* *p* < .05. ***p* < .01.

## Appendix B: Moderation Analysis

**Table B1.** Moderation Analysis.

Model 1: variable	β	*t*	*p*-Value	LLCI	ULCI
Internalizing symptoms	.22	3.67	.0000	6.02	6.64
Parent strengths	.10	1.89	.059	−0.00	0.21
Interaction	−.02	1.01	.30	−0.06	0.21
Model 2: variable					
Internalizing symptoms	.19	3.26	.001	0.07	0.30
Child strengths	.27	2.96	.003	0.09	0.45
Interaction	.08	2.19	.03	0.00	0.15
